# SN algorithm: analysis of temporal clinical data for mining periodic patterns and impending augury

**DOI:** 10.1186/2043-9113-3-24

**Published:** 2013-11-28

**Authors:** Dipankar Sengupta, Pradeep K Naik

**Affiliations:** 1 Department of Biotechnology and Bioinformatics, Jaypee University of Information Technology, Waknaghat, Solan, H.P, India

**Keywords:** Association rule mining, Clinical informatics, Data mining, Jacobian, Jacobian determinant, Temporal

## Abstract

**Background:**

EHR (Electronic Health Record) system has led to development of specialized form of clinical databases which enable storage of information in temporal prospective. It has been a big challenge for mining this form of clinical data considering varied temporal points. This study proposes a conjoined solution to analyze the clinical parameters akin to a disease. We have used “association rule mining algorithm” to discover association rules among clinical parameters that can be augmented with the disease. Furthermore, we have proposed a new algorithm, SN algorithm, to map clinical parameters along with a disease state at various temporal points.

**Result:**

SN algorithm is based on Jacobian approach, which augurs the state of a disease ‘S_n_’ at a given temporal point ‘T_n_’ by mapping the derivatives with the temporal point ‘T_0_’, whose state of disease ‘S_0_’ is known. The predictive ability of the proposed algorithm is evaluated in a temporal clinical data set of brain tumor patients. We have obtained a very high prediction accuracy of ~97% for a brain tumor state ‘S_n_’ for any temporal point ‘T_n_’.

**Conclusion:**

The results indicate that the methodology followed may be of good value to the diagnostic procedure, especially for analyzing temporal form of clinical data.

## Background

Advancement in clinical research and diagnostic processes produce large amount of data that are heterogeneous in nature [1]. The data obtained from a patient generally include patient complaints, history, clinical symptoms and signs, physician’s examinations, biochemical analyses, imaging profiles, pathologies, therapies and other measurements [2] pertaining to clinical diagnostics. Since there is lack of integration of these data, the importance and relationships among the clinical parameters pertaining to occurrence of diseases is difficult to analyze. Immense efforts have been made recently to address the issues concerning to extract information from these heterogeneous clinical data. Henceforth, development of novel informatics techniques based on mathematical or statistical models are essential. This development will provide a better understanding of the complex nature of diseases and guide in more accurate & improved diagnosis for better therapies.

Path breaking step in the field of clinical informatics was the development of EHR/EMR (Electronic Health/Medical Records) which led to evolution of information technology in the field of clinical sciences [3]. As an effort to facilitate access to this wealth of information, data warehouses were developed that contained clinical data from healthcare organizations [4]. The enormous amount of data collected by EHR/EMR provides additional value when integrated and stored in data warehouses suitable for data mining techniques such as co-occurrence analysis and association mining. As an archetype, National Cancer Institute has developed a medical knowledge information system integrated with data mining applications [5]. Similarly, New York-Presbyterian Hospital is using an electronic health record system for the past several years and maintaining a longitudinal record for each patient [6]. Congruous mining techniques such as co-occurrence statistics analyzes the importance of clinical data associations together systematically rather than random combinations [5]. Similarly, technique using association rule mining is a general purpose rule discovery scheme and has been widely used for discovering rules based on the importance of finding disease co-occurrences.

Crucial to mining in clinical informatics is to use background knowledge to discover interesting interpretable and non-trivial relationships, to construct rule-based and other symbolic-type models that can be reviewed and scrutinized by experts, to discover models that offer an explanation when used for prediction and, also to bridge model discovery and decision support to deploy predictive models in daily clinical practice [7]. Among the various mining approaches, predictive data mining approach is gaining impulse among the researchers and clinical practitioners as it utilizes the knowledge available in the clinical domain and explains proposed decision for the proposed model [7]. The goal of predictive data mining in clinical medicine is to derive models that can use patient specific information to predict the outcome of interest and thereby support clinical decision-making [7]. Among the various approaches, Naive Bayesian classifier is one of the earliest designed approach that is based on probability. It is one of the simplest yet a useful and often a fairly accurate predictive data mining method. However, since it is dependent on the type of data subjected to mining, it may be inclined in case of biased clinical data set [8]. Another popular data mining technique is decision tree which is based on recursive data partitioning, where in each iteration the data is split according to the value of a selected clinical attribute. However, its performance is impacted because of clinical data segmentation [9]. Logistic regression is another powerful and well-established statistical method used in predictive clinical mining. It is an extension of normal regression method that models a two-valued outcome for occurrence or non-occurrence of some event. It is based on multiplicative probability model that utilizes maximum likelihood estimation to determine the coefficients in its probability formula. Handling of the missing values usually causes problem in this approach [10]. For a long period artificial neural network models were the most popular artificial intelligence-based predictive algorithm used in clinical medicine. Albeit they have a number of deficiencies that include high sensitivity to the parameters of the method - including those that determine the architecture of the network and induction of the model that may be hard to interpret by domain experts [11]. Support vector machines (SVM) are perhaps today’s most powerful classification algorithm in terms of predictive accuracy and most popular in clinical informatics. However, the exception are linear kernels, where the structure of the model can be easily revealed through the coefficients that define a linear hyperplane, and it use a formalism that is often unsuitable for interpretation by human experts [12].

An interesting prospective in these predictive mining of heterogeneous clinical data would be an approach that could analyse the temporal form. The discovery of hidden periodic patterns in temporal data, apart from unveiling important information, can facilitate data management substantially [13]. However, very limited work has been done so far on data mining of temporal data, which demonstrates generalization of pattern mining in time-series data [14]. For instance, we can model the change of climatic conditions in a spatial region as a sequence of existing or a past set of values. Periodicity has only been studied in the context of temporal analysis of time-series based databases that addressing the following problem: given a long sequence S and a period T, the aim is to discover the most representative trend that repeats itself in S every T timestamps [15]. This uses a tree structure to count the support of multiple patterns at two database points and comparatively studies the problem of finding sets of events that appear together periodically [16]. However, it does not take into consideration the order of occurrence of events. Whereas, in case of temporal clinical data it is necessary to consider specific order of occurrence of events that are associated with the state of a disease. Considering the given scenario, SN algorithm proposed in this study, is a novel predictive data mining algorithm based on Jacobian approach. It will traverse selective clinical parameters at different temporal points to augur the possible “STATE” of the disease. The advantage of this algorithm over existing predictive techniques like logistic regression or ANN or SVM is that it is independent of coefficients for prediction. Moreover, it keeps a track of previous versus new information i.e. for a given patient it predicts the corresponding state of the disease based on the value of input clinical parameters along with the state of the disease at previous temporal point.

In this study we have defined the temporal mining problem of clinical data in terms of (a) discovery of associative rules for clinical parameters, which can be associated with a specific disease (clinical parameters are discovered by apriori association mining); and (b) an algorithm for traversing the clinical parameters of temporal points ‘T’ (T_0_, T_1_ … T_n_) in order of their occurrences, alongwith mapping the values observed for each point with the previous one. This helps in auguring the state of a specific disease at point T_n_ whose result is unknown. To predict the state of a disease at point T_n_, we propose a new algorithm (we termed it as ‘SN algorithm’) based on Jacobian transformation by considering different temporal points, in which Jacobian of selected clinical parameters are associated with the state of that disease. Hence, derivatives ‘J’ (J_0_, J_1_…) of temporal points ‘T’ (T_0_, T_1_ …) along with respective states ‘S’ (S_0_, S_1_....) are mapped with a future point (T_n_) Jacobian (J_n_) and finally its determinant (J”) is calculated to obtain a possible state (S_n_).

## Methods

### Data warehouse development

Approval was obtained from joint institutional review board of hospitals under Indira Gandhi Medical College, Himachal Pradesh, India (IGMC Study Approval No.: HFW(MC-II)G-7/07-Vol. IV-17754) and patient consent was taken for using the clinical data. Clinical data for all the human subjects have been analyzed anonymously. Based on NOC (No objection certificate) received from the hospitals in India, all the patient information was received corresponding to IDs (Identification Number). There is no disclosure of the hospital names or patient information in this study.

An in-house data warehouse was developed using MySQL (v5.019) to store the clinical data collected from various Government Hospitals across India. By nature this data is heterogeneous and obtained in different forms, such as printed & manual reports, doctor’s advice & prescription, images in form of CT scan, MRI, etc. As there are no EHR/EMR system implemented in these hospitals, data were collected in form of hard copies and then manually entered into the electronic form. Accuracy of data is an important criteria to be considered during development of a clinical warehouse especially when there are no EHR/EMR implemented [17]. Data incorrectness usually exists because of design or operational deficiency and can be identified where the mapping between the information system state and the real world state break down [17]. Henceforth, with utmost care the dimensional model (data model) of the clinical warehouse was designed based on the descriptive and measurable features of the clinical data [18]. Further, it consists of date and time dimension that ensures temporal storage of data for a patient. Also, to check the operational deficiencies, the quality assurance of data was ensured by implementing appropriate data processing codes for range and data validation checks [19], re-entering samples of data to assess for accuracy, checks for data completeness and attention for data consistency [20].

The warehouse is integrated with the data mining process for analysis. Data were preprocessed, normalized based on prescribed clinical ranges [19] and analyzed for identification of associative clinical rules for disease. The parameters identified to be associated with the state of the disease were used to map at different temporal points based on SN algorithm which in turns help in auguring the state of the disease.

### Association mining

This study focuses on identification of clinical parameters that can be associated with progressive state of a disease by implementing association mining algorithm. It is a popular data mining technique [21] that tries to find interesting patterns in large databases [22]. The Apriori algorithm exploits the downward closure property, which states that if an item set is infrequent, all of its supersets must be infrequent too. The classic framework for association rule mining uses *support* and *confidence* as thresholds for constraining the search space. Each item set has an associated statistical measure called *support*. For an itemset X ⊂ I, support(X) = s, if the fraction of transactions in the dataset D containing X = s [23]. The *confidence* of an association rule X = > Y in *D* is the conditional probability of having Y contained in a transaction, given that X is contained in that transaction: confidence (X= > Y) = P (Y|X) = support(XY)/support(X) [22]. A *confidence value* of 100 for a certain rule means that the possibility of obtaining outcome Y when X is a given condition (X → Y) is 100%; if not, the possibility of A → B is defined as a value (possible rule) between 0 and 100.

It is arduous to predispose appropriate criteria for any two parameters in association rule mining, because information is obtained based on a minimum threshold for support and confidence [22]. As such, in this study, the frequent item sets were discovered based upon selected parameters for preprocessed clinical dataset that were subjected to confidence of atleast 50%, when the minimum support was defined to 30%. STATISTICA DATAMINER 9.1 [24] was used to calculate the frequency of each item set with support% criteria of at least 30 along with head and body iteration rate of 10. All frequent item sets obtained were subjected for the discovery of association rules. The final confidence to deduce rule was set to at least 85% through a physician’s opinion and the process was executed with antecedent and precedent iteration rate of value 10.

### SN algorithm

The proposed SN algorithm is being designed for traversing across the clinical measures of a patient pertaining to particular disease at varied temporal points and augur the possible “STATE” of that disease. The state of temporal point ‘T_n_’ is obtained as Jacobian determinant for cross product of derivatives of selected clinical parameters for ‘T_n_’ and its immediate predecessor point. The clinical parameters are selected for a disease based on the associative rules deciphered above. The selected clinical parameters acts as base point for SN algorithm to extrapolate the progression of disease at given time point ‘T_n_’. In detail the algorithm consists of following four steps:

i. With an input of set of temporal points (T_0_, T_1_, T_2_,....,T_n_), a set of selected clinical parameter values (P_0_, P_1_, P_2_,…, P_n_) for a patient along with the state of disease (S_0_, S_1_, S_2_,…,S_n_) is chosen for each temporal point, where State ‘S_n_’ is unknown for the point T_n_.

ii. Jacobian transformation is applied over the set of selected parameters (P_0_, P_1_, P_2_,…, P_n_) for each of the temporal point ‘T’ to obtain the Jacobian.

iii. Jacobian (J_0_, J_1_, J_2_, …, J_n_) for each temporal point along with state of disease ‘S’ is then mapped to the values of other temporal point.

iv. Jacobian determinant (J”) is then determined based on the mapping done in step iii for predicting augury of state S_n_ for point T_n_.

Mathematically, Jacobian, mapping of Jacobian in time-space as area and estimation of its determinant for area can be explained as follows [25].

Let T (u, v) be a smooth coordinate transformation with Jacobian J (u,v) and let R be the rectangle spanned by du = (du, 0) and dv = (0, dv). If du and dv are sufficiently close to 0, then T (R) is approximately the same as the parallelogram spanned by:

$$\begin{array}{l} \text{dx}=J\;\left(u,v\right)\;\text{du}=\left(x_{u}\text{du},y_{u}\text{du},0\right)\\ \text{dy}=J\;\left(u,v\right)\;\text{dv}=\left(x_{v}\text{dv},y_{v}\text{dv},0\right) \end{array}$$

Let dA denote the area of the parallelogram spanned by dx and dy parameter, then dA approximates the area of T (R) for du and dv sufficiently close to 0.

The cross product of dx & dy is given as,

$$\text{dx}*\text{dy}=<0,0\left|{}_{\mathrm{yu}}^{\text{xu}}{\;\text{yv}}^{\;\text{xv}}\right|>\text{dudv}$$

from which the differential area *dA* can be obtained as:

$$\text{dA}=\left|\frac{\partial \left(x,y\right)}{\partial \left(u,v\right)}\right|\;\text{dudv}$$

**Figure 1:**
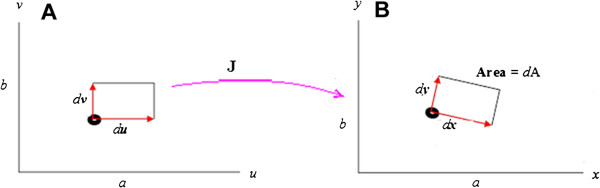
**Area differential approach based on Jacobian transformation.** (**A** - Temporal point 1; **B** - Temporal point 2).

Area of a small region in the uv-plane is scaled by Jacobian determinant to approximate areas of small images in the xy-plane (Figure 1).

**Figure 2:**
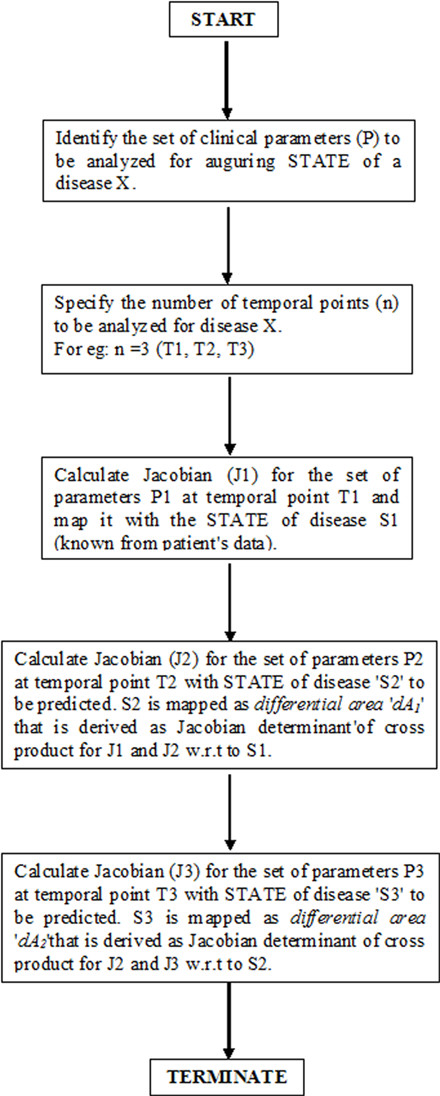
Flow diagram of SN algorithm.

The flow-diagram in Figure 2 depicts the methodology of SN algorithm in a logical representation.

## Result & discussion

To test the predictability of SN algorithm we have taken a temporal case study of 55 patients suffering from brain tumor over a period of 6 months. The clinical data was collected from various Government Hospitals in India and stored in the in-house developed data warehouse. The data mining process involves two stages. In the first phase, brain tumor was treated as a response and was analyzed corresponding to investigating parameters of blood analysis, KFT (Kidney Functionality Test), LFT (Liver Functionality Test), sugar level, triplets of blood pressure and MRI/CT scan images. Association rule mining was applied to this dataset using STATSTICA DATAMINER. The set of rules deciphered from association mining (included in Table 1) with 85% confidence and atleast 50% support criteria suggests that Creatinine ‘c’, Blood Urea Nitrogen (BUN) ‘b’, SGOT (Serum Glutamic Oxaloacetic Transaminase) ‘s’ and SGPT (Serum Pyruvic Transaminase) ‘g’ are the clinical diagnostic parameters which can be associated with occurrence of brain tumor in patients [26].

**Table 1:** Association rules mined for various diagnostic parameters that are associated with the occurrence of brain tumor in patients

**Association rule**	**Support%**	**Confidence%**	**Correlation%**
KFT_Creatinine = HIGH == > KFT_BUN = HIGH	56.75	100	77.45
KFT_Creatinine = HIGH == > STATE = 1	56.75	100	77.77
KFT_BUN = HIGH == > STATE = 1	78.37	85.29	90.8
KFT_Creatinine = HIGH, KFT_BUN = HIGH == > STATE = 1	56.75	100	79.77
LFT_SGOT = HIGH == > STATE = 1	62.16	98.83	81.72
LFT_SGOT = HIGH == > LFT_SGPT = HIGH, STATE = 1	62.16	95.83	85.71
LFT_SGPT = HIGH == > STATE = 1	81.08	88.23	89.56
Haemoglobin_content = NORMAL == > STATE = 1	59.45	100	81.64

Support%, confidence% and correlation% for various combinations of parameter sets are included.

In the second phase of the study, SN algorithm was applied over 3 temporal state points T(T1, T2, T3) for each patient P(P1, P2,…P55) in which state S_o_(S1, S2, S3) of the disease at each temporal point was considered along with the values for Creatinine c(c1, c2, c3), BUN b(b1, b2, b3), SGOT s(s1, s2, s3) and SGPT g(g1, g2, g3) parameters as depicted in Table 2. Observed State S’_o_(S’1, S’2, S’3) pertaining to each temporal point T(T1, T2, T3) for each patient were determined based on CT/MRI results and diagnosis/recommendation of oncologist. Certainty of the algorithm has being analyzed by the accuracy factor that is based on the observed state “S’_o_” and Predicted State “S_o_”.

**Table 2:** Temporal points along with various selected clinical parameters corresponding to brain tumor

P1, T1, c1, b1, s1, g1	P2, T1, c’1, b’1, s’1, g’1	…	P55, T1, c”1, b”1, s”1, g”1
P1, T2, c1′, b1′, s1′, g1′	P2, T2, c’2′, b′2′, s’2′, g’2′	…	P55, T2, c”2′, b”2′, s”2′, g”2′
P1, T3, c1″, b1″, s1″, g1″	P2, T3, c’3″, b’3″, s’3″, g’3″	…	P55, T3, c”3″, b”3″, s”3″, g”3″

L (c, b, s, g) is the transformation with Jacobian J (c, b, s, g) applied for each predicted state S_o_(S1, S2, S3). Jacobian is calculated for each of the functional parameter (c,b,s,g) of the first temporal point T1 which is mapped with the state S1 (S’1 is selected to map the initial state of disease at first temporal point i.e. S1 = S’1) as area curve. J1 (c1,b1,s1,g1) is the Jacobian for patient ’P1′ at time ‘T1’ that is mapped to the state of the disease ‘S1′. Similarly for the second temporal point ‘T2′, Jacobian J2 (c1′, b1′, s1′, g1′) is to be mapped with S2 (represented by area *dA*) for patient ‘P1′. Based on the cross product of Jacobian for point T1 and T2, the differential area ‘*dA’* is mapped as Jacobian determinant to obtain S2 state. The accuracy of predicted state S2 based on SN algorithm was 100% when compared to observed S’2 state (Additional file 1). However, for the third temporal point only Jacobian J3 (c1”, b1”, s1”, g1”) for the parameters was obtained and S’3 result was in a hidden state. To obtain the S3 predicted state, differential area was mapped as Jacobian determinant based on cross products of Jacobian for points T2 and T3. Predictability of the S3 state with the hidden S’3 state was 92.7% accurate (Additional file 1). Thus, the proposed algorithm is helping in auguring the state of disease for brain tumor patients, independent of results from MRI, CT scan, arteriogram or small dime craniotomy based on temporal values for Creatinine, BUN, SGOT & SGPT clinical parameters.

Analyzing the time complexity of the proposed SN algorithm will be essential to evaluate its robustness. Master method is been applied to estimate the time complexity which can be associated with proposed SN algorithm. The time complexity has been calculated in terms of Big O notation given as:

$$T\left(n\right)=O\left(f\left(n\right)\right)$$

The expected running time (d) for this algorithm is directly dependent to number of sub-problems (a) to be analyzed which is the number of temporal points for a particular case, considering the shrinkage factor (b) to be greater than 1. Henceforth, for the given algorithm, as observed: a = b^d^, the time complexity associated with the algorithm can be estimated as:

O=nlogn

Where, n - > number of temporal points analyzed for given set of parameters.

The estimated time complexity of the proposed SN algorithm suggests minimal execution time for auguring the STATE of disease at a particular temporal point. However, increasing the number of temporal point will directly proportionate the execution time.

## Conclusion

In this study, temporal mining problem associated with clinical data was raised as a research problem, corresponding to which SN algorithm has been proposed. The algorithm is based on Jacobian and mapping of its derivative as area. The accuracy of the algorithm was evaluated using a data set of 55 patients suffering from brain tumor. Using this algorithm we have achieved 100% accuracy in predicting the progression of brain tumor at 2nd temporal point by mapping with the Jacobian derivative of 1st temporal point. In contrast, we have predicted the disease progression with an accuracy of 92.7% at 3rd temporal point based on 2nd temporal point. Taken together, the algorithm developed in this study hold a great potential in monitoring the state of disease based on regular input values for minimal set of clinical parameters. However, the effectiveness of the algorithm needs to be further evaluated by analyzing the parameters associated with other diseases and analyzing it over various temporal points for a group of patients.

## Consent

Written informed consent was obtained from the patient for the publication of this report and any accompanying images.

## Abbreviations

ANN: Artificial neural network; BUN: Blood urea nitrogen; CT: Computed tomography; EHR/EMR: Electronic health/medical record; ID: Identification number; KFT: Kidney functionality test; LFT: Liver functionality test; MRI: Magnetic resonance imaging; NOC: No objection certificate; SGOT: Serum glutamic oxaloacetic transaminase; SGPT: Serum pyruvic transaminase; SVM: Support vector machine.

## Competing interests

No competing interests to be declared. No current external funding sources for this study.

## Authors’ contributions

DS - Design of study, collection of data, designing of SN algorithm, testing the algorithm on specific case study, analysis of results, drafting of manuscript. PKN - Design of study, collection of data, analysis of results, finalizing the draft manuscript. Both authors read and approved the final manuscript.

## Supplementary Material

Additional file 1 Prediction at Temporal Point T2, Prediction at Temporal Point T3. Click here for file
