# Supplementary material for: SN algorithm: analysis of temporal clinical data for mining periodic patterns and impending augury
Source: J Clin Bioinforma. 2013 Nov 28;3:24. doi: 10.1186/2043-9113-3-24 (PMC4177143; doi:10.1186/2043-9113-3-24)
Supplement: Additional file 1 — Prediction at Temporal Point T2, Prediction at Temporal Point T3. [file 2043-9113-3-24-S1.docx]

**Prediction at Temporal Point T2**

| **Patient_ID** | **State T1**  (Known from Patient Data) | **Actual State for T2** (Known from Patient Data) | **Predicted State for T2** (Calculated via SN algorithm) | **Prediction Status** |
| --- | --- | --- | --- | --- |
| 1 | 1.0 | 1.3 | 1.26 | Correct |
| 2 | 1.0 | 1.3 | 1.26 | Correct |
| 3 | 1.0 | 1.4 | 1.43 | Correct |
| 4 | 1.0 | 0.9 | 0.91 | Correct |
| 5 | 1.0 | 1.2 | 1.22 | Correct |
| 6 | 1.0 | 1.3 | 1.29 | Correct |
| 7 | 1.0 | 1.5 | 1.48 | Correct |
| 8 | 1.0 | 0.8 | 0.78 | Correct |
| 9 | 1.0 | 0.7 | 0.69 | Correct |
| 10 | 1.0 | 0.9 | 0.92 | Correct |
| 11 | 1.0 | 0.7 | 0.69 | Correct |
| 12 | 1.0 | 0.8 | 0.81 | Correct |
| 13 | 1.0 | 0.9 | 0.92 | Correct |
| 14 | 1.0 | 1.3 | 1.29 | Correct |
| 15 | 1.0 | 1.6 | 1.64 | Correct |
| 16 | 1.0 | 1.6 | 1.62 | Correct |
| 17 | 1.0 | 1.5 | 1.54 | Correct |
| 18 | 1.0 | 1.3 | 1.29 | Correct |
| 19 | 1.0 | 1.1 | 1.07 | Correct |
| 20 | 1.0 | 1.0 | 1.03 | Correct |
| 21 | 1.0 | 1.2 | 1.20 | Correct |
| 22 | 1.0 | 1.0 | 1.02 | Correct |
| 23 | 1.0 | 1.0 | 1.04 | Correct |
| 24 | 1.0 | 1.4 | 1.39 | Correct |
| 25 | 1.0 | 1.3 | 1.29 | Correct |
| 26 | 1.0 | 0.9 | 0.88 | Correct |
| 27 | 1.0 | 0.7 | 0.72 | Correct |
| 28 | 1.0 | 0.7 | 0.69 | Correct |
| 29 | 1.0 | 0.7 | 0.69 | Correct |
| 30 | 1.0 | 1.3 | 1.29 | Correct |
| 31 | 1.0 | 1.2 | 1.23 | Correct |
| 32 | 1.0 | 1.5 | 1.51 | Correct |
| 33 | 1.0 | 1.4 | 1.42 | Correct |
| 34 | 1.0 | 1.3 | 1.31 | Correct |
| 35 | 1.0 | 1.1 | 1.09 | Correct |
| 36 | 1.0 | 1.1 | 1.11 | Correct |
| 37 | 1.0 | 1.1 | 1.09 | Correct |
| 38 | 1.0 | 0.7 | 0.69 | Correct |
| 39 | 1.0 | 0.8 | 0.81 | Correct |
| 40 | 1.0 | 0.8 | 0.81 | Correct |
| 41 | 1.0 | 1.3 | 1.32 | Correct |
| 42 | 1.0 | 0.8 | 0.78 | Correct |
| 43 | 1.0 | 0.9 | 0.93 | Correct |
| 44 | 1.0 | 1.4 | 1.38 | Correct |
| 45 | 1.0 | 1.4 | 1.39 | Correct |
| 46 | 1.0 | 1.4 | 1.39 | Correct |
| 47 | 1.0 | 1.4 | 1.41 | Correct |
| 48 | 1.0 | 1.5 | 1.51 | Correct |
| 49 | 1.0 | 1.2 | 1.23 | Correct |
| 50 | 1.0 | 0.7 | 0.73 | Correct |
| 51 | 1.0 | 0.8 | 0.81 | Correct |
| 52 | 1.0 | 0.8 | 0.81 | Correct |
| 53 | 1.0 | 0.9 | 0.89 | Correct |
| 54 | 1.0 | 1.0 | 0.99 | Correct |
| 55 | 1.0 | 1.0 | 0.99 | Correct |

Predicted State is being represented till 2nd decimal point. A **5% margin** is being considered in assessing the prediction status by comparing Actual and Predicted T2 state.

**No. of True Positives (Correct Predictions) for T2 state =** 55

**No. of False Negatives (Incorrect Predictions) for T2 state =** 0

**Accuracy for T2 state =** 100%

**Prediction at Temporal Point T3**

| **Patient_ID** | **State T2**  (From T2 prediction) | **Actual State for T3** (Known from Patient Data) | **Predicted State for T3** (Calculated via SN algorithm) | **Prediction Status** |
| --- | --- | --- | --- | --- |
| 1 | 1.26 | 1.4 | 1.43 | Correct |
| 2 | 1.26 | 1.5 | 1.47 | Correct |
| 3 | 1.43 | 1.4 | 1.4 | Correct |
| 4 | 0.91 | 0.8 | 0.77 | Correct |
| 5 | 1.22 | 1.1 | 1.09 | Correct |
| 6 | 1.29 | 1.0 | 1.04 | Correct |
| 7 | 1.48 | 1.4 | 1.39 | Correct |
| 8 | 0.78 | 0.8 | 0.78 | Correct |
| 9 | 0.69 | 0.7 | 0.68 | Correct |
| 10 | 0.92 | 0.9 | 0.91 | Correct |
| 11 | 0.69 | 0.6 | 0.61 | Correct |
| 12 | 0.81 | 0.7 | 0.72 | Correct |
| 13 | 0.92 | 0.8 | **0.87** | **Incorrect** |
| 14 | 1.29 | 1.2 | 1.19 | Correct |
| 15 | 1.64 | 1.6 | 1.57 | Correct |
| 16 | 1.62 | 1.5 | 1.51 | Correct |
| 17 | 1.54 | 1.4 | 1.39 | Correct |
| 18 | 1.29 | 1.3 | 1.28 | Correct |
| 19 | 1.07 | 1.0 | **0.92** | **Incorrect** |
| 20 | 1.03 | 1.0 | 1.02 | Correct |
| 21 | 1.20 | 1.1 | 1.09 | Correct |
| 22 | 1.02 | 0.8 | 0.83 | Correct |
| 23 | 1.04 | 0.8 | 0.84 | Correct |
| 24 | 1.39 | 1.3 | 1.29 | Correct |
| 25 | 1.29 | 1.3 | 1.29 | Correct |
| 26 | 0.88 | 0.8 | 0.81 | Correct |
| 27 | 0.72 | 0.7 | 0.71 | Correct |
| 28 | 0.69 | 0.7 | 0.7 | Correct |
| 29 | 0.69 | 0.6 | 0.58 | Correct |
| 30 | 1.29 | 1.2 | 1.19 | Correct |
| 31 | 1.23 | 1.2 | 1.21 | Correct |
| 32 | 1.51 | 1.4 | 1.42 | Correct |
| 33 | 1.42 | 1.3 | 1.31 | Correct |
| 34 | 1.31 | 1.1 | 1.08 | Correct |
| 35 | 1.09 | 0.8 | 0.83 | Correct |
| 36 | 1.11 | 1.0 | 1.02 | Correct |
| 37 | 1.09 | 1.3 | **1.36** | **Incorrect** |
| 38 | 0.69 | 0.6 | 0.61 | Correct |
| 39 | 0.81 | 0.7 | 0.69 | Correct |
| 40 | 0.81 | 0.7 | **0.76** | **Incorrect** |
| 41 | 1.32 | 1.3 | 1.29 | Correct |
| 42 | 0.78 | 0.9 | 0.87 | Correct |
| 43 | 0.93 | 0.9 | 0.94 | Correct |
| 44 | 1.38 | 1.2 | 1.17 | Correct |
| 45 | 1.39 | 1.2 | 1.24 | Correct |
| 46 | 1.39 | 1.3 | 1.27 | Correct |
| 47 | 1.41 | 1.1 | 1.13 | Correct |
| 48 | 1.51 | 1.4 | 1.39 | Correct |
| 49 | 1.23 | 1.0 | 0.98 | Correct |
| 50 | 0.73 | 0.6 | 0.57 | Correct |
| 51 | 0.81 | 0.7 | 0.69 | Correct |
| 52 | 0.81 | 0.7 | 0.69 | Correct |
| 53 | 0.89 | 0.9 | 0.87 | Correct |
| 54 | 0.99 | 1.0 | 1.01 | Correct |
| 55 | 0.99 | 1.0 | 1.01 | Correct |

Predicted State is being represented till 2nd decimal point. A **5% margin** is being considered in assessing the prediction status by comparing Actual and Predicted T2 state.

**No. of True Positives (Correct Predictions) for T3 state =** 51

**No. of False Negatives (Incorrect Predictions) for T3 state =** 04

**Accuracy for T3 state =** 92.7%

**Overall Accuracy observed (T2 and T3) = 96.35% ~ 97%**
